# 1620. Identification of plasma biomarkers of neurological complications in patients with congenital cytomegalovirus infection by proteome analysis

**DOI:** 10.1093/ofid/ofad500.1455

**Published:** 2023-11-27

**Authors:** Makoto Yamaguchi, Takako Suzuki, Hiroyuki Kidokoro, Ken-ichi Iwada, Yuto Fukuda, Kazunori Haruta, Yuka Torii, Yoshinori Ito, Jun-ichi Kawada

**Affiliations:** Nagoya University Graduate School of Medicine, Nagoya, Aichi, Japan; Nagoya University Graduate School of Medicine, Nagoya, Aichi, Japan; Nagoya University Graduate School of Medicine, Nagoya, Aichi, Japan; Nagoya University Graduate School of Medicine, Nagoya, Aichi, Japan; Nagoya University Graduate School of Medicine, Nagoya, Aichi, Japan; Nagoya University Graduate School of Medicine, Nagoya, Aichi, Japan; Nagoya University Graduate School of Medicine, Nagoya, Aichi, Japan; Nihon University School of Medicine, Itabashi-ku, Tokyo, Japan; Nagoya University Graduate School of Medicine, Nagoya, Aichi, Japan

## Abstract

**Background:**

Congenital cytomegalovirus infection (cCMV) is the leading cause of non-hereditary sensorineural hearing loss and can cause other long-term neurodevelopmental disabilities. To consider antiviral treatment, it is important to differentiate between symptomatic and asymptomatic patients by early infancy. However, it is difficult to identify neurological complications in some cases. This study aimed to identify candidate plasma biomarkers for neurological complications of cCMV during the neonatal period using proteome analysis.

**Methods:**

This study retrospectively enrolled five patients with asymptomatic cCMV and nine patients with symptomatic cCMV, including those with hearing loss and/or abnormal brain imaging. Plasma samples were obtained during the neonatal period. After extraction of 14 high-abundant proteins from the plasma, proteins were digested by trypsin. The peptides were analyzed by liquid chromatography–mass spectrometry using an Orbitrap Fusion mass spectrometer. Protein quantification was performed using Proteome Discoverer. Levels of differently expressed proteins were validated by enzyme-linked immunosorbent assay.

**Results:**

In total, 456 proteins were identified and quantified. Levels of 65 proteins were significantly higher in patients with symptomatic cCMV than in those with asymptomatic cCMV. The top two enriched categories for these proteins according to pathway enrichment analysis were “complement and coagulation cascade” and “inflammatory response.” In contrast, levels of 30 proteins were significantly higher in patients with abnormal brain imaging. Plasma levels of fms-related receptor tyrosine kinase 4 (FLT4) were significantly higher in patients with symptomatic cCMV than in those with asymptomatic cCMV. Moreover, levels of plasma peptidylprolyl isomerase A (PPIA) were significantly higher in patients with abnormal brain imaging than in those without.

**Conclusion:**

Proteome analysis showed that the proteins related to inflammatory response pathways were upregulated in patients with symptomatic cCMV. FLT4 and PPIA can be used as novel diagnostic biomarkers for neurological complications of cCMV to enable early therapeutic intervention with antiviral agents.

**Disclosures:**

**All Authors**: No reported disclosures

